# Transferability of the PRS estimates for height and BMI obtained from the European ethnic groups to the Western Russian populations

**DOI:** 10.3389/fgene.2023.1086709

**Published:** 2023-01-16

**Authors:** E. A. Albert, O. A. Kondratieva, E. E. Baranova, O. V. Sagaydak, M. S. Belenikin, G. Y. Zobkova, E. S. Kuznetsova, A. A. Deviatkin, A. A. Zhurov, E. A. Karpulevich, P. Y. Volchkov, M. V. Vorontsova

**Affiliations:** ^1^ National Medical Research Center for Endocrinology, Moscow, Russia; ^2^ Life Sciences Research Center, Moscow Institute of Physics and Technology, Dolgoprudniy, Russia; ^3^ Department of Information Systems, Ivannikov Institute for System Programming of the Russian Academy of Sciences, Moscow, Russia; ^4^ Evogen LLC, Moscow, Russia

**Keywords:** PRS, GWAS summary statistic, population structure, BMI, height

## Abstract

Genetic data plays an increasingly important role in modern medicine. Decrease in the cost of sequencing with subsequent increase in imputation accuracy, and the accumulation of large amounts of high-quality genetic data enable the creation of polygenic risk scores (PRSs) to perform genotype–phenotype associations. The accuracy of phenotype prediction primarily depends on the overall trait heritability, Genome-wide association studies cohort size, and the similarity of genetic background between the base and the target cohort. Here we utilized 8,664 high coverage genomic samples collected across Russia by “Evogen”, a Russian biomedical company, to evaluate the predictive power of PRSs based on summary statistics established on cohorts of European ancestry for basic phenotypic traits, namely height and BMI. We have demonstrated that the PRSs calculated for selected traits in three distinct Russian populations, recapitulate the predictive power from the original studies. This is evidence that GWAS summary statistics calculated on cohorts of European ancestry are transferable onto at least some ethnic groups in Russia.

## 1 Introduction

Unrelenting progress in sequencing technologies has led to an unprecedented accumulation of a vast amount of “omics” data. Although the era of human genomics began two decades ago, this field has remained a booming scientific and medical area with many knowledge gaps remaining. Initial versions of the human genome were published in 2001 (Lander et al., 2001; Venter et al., 2001), but a complete sequencing was performed only twenty years later (Nurk et al., 2022). Data accumulation continues while genomic sequences are increasingly used in practical medicine.

The organism of each human is unique and inimitable. This is primarily predetermined by differences in our genomic sequences (Varki et al., 2008). Such variations create the need for personalized medicine, a search for the optimal way to manage a specific pathology for a concrete patient. For timely diagnosis and therapy, optimal selection is crucial to predict disease risk. Genome-wide association studies (GWAS) produce estimates for patients separated into different cohorts with varying risks for developing a disease according to their genomic information. GWAS studies could reveal single nucleotide polymorphisms (SNPs) significantly associated with scrutinized traits. However, most SNPs are omitted during GWAS analysis due to the absence of significance, whereas taking into account all available SNPs provides a better explanation for the association between genetics and the selected trait (Yang et al., 2010).

The contribution of genetic factors to pathogenesis in a wide variety of human diseases is well known (Rebbeck, 2017; Verheijen and Sleegers, 2018; Kwon et al., 2019; Schoettler et al., 2019; Graham and Xavier, 2020; Mikhaylenko et al., 2020). Nevertheless, the degree of genetic impact in each particular pathological condition might be hard to estimate. In cases with unclear genetic influence, one should consider the involvement of several genes in the development of the pathology. Multiple genetic markers (e.g., SNPs) could be combined into a single score for anticipating disease risk (Dudbridge, 2013). Polygenic risk score (PRS) is an approach for predicting personal predisposition to a given disease (Lambert et al., 2019). PRS could be calculated by summarizing an individual’s risk alleles, normalized according to the weight of risk allele size effect (Choi et al., 2020). PRSs may improve current clinical risk prediction models for many diseases, such as breast cancer, prostate cancer, coronary artery disease, obesity, type 1 diabetes, type 2 diabetes, and Alzheimer’s disease—all reviewed by Lambert et al. (2019) . In combination with clinical risk data, PRS could become an important tool for precision medicine. Patients with higher polygenic risks need to undergo more intensive diagnostic procedures than patients with lower risks (Torkamani et al., 2018). Additionally, some treatments for one disease could induce the onset of another. For example, statins prescribed to prevent strokes preclude less than two strokes out of one hundred, while provoking diabetes development in one out of one hundred cases (Torkamani et al., 2018). The use of PRS could provide more efficient and informed treatment of a particular disease, for example CAD (Klarin and Natarajan, 2022) or schizophrenia (Binder, 2019).

Based on their genome, humans can be divided into various ethnic groups with different predispositions to pathologies. Large-scale genetic studies on human diseases are mostly based on data collected from Europeans. As a result, the knowledge about possible genomic variation is biased toward the specific background population (Sirugo et al., 2019). 86.1% of GWAS participants originate from four countries: United Kingdom (40.5%), United States (19.8%), Japan (14.3%), and Iceland (11.5%). However, the European superpopulation can be separated into several groups (Nelis et al., 2009). For example, the Russian population is distinct from the British cluster (Nelis et al., 2009; Zhernakova et al., 2020; Oleksyk et al., 2021). To the best of our knowledge, the most representative study of Russian population genetics by whole genome sequencing included just 264 persons (Mallick et al., 2016; Pagani et al., 2016; Zhernakova et al., 2020), and the largest genotyping studies included hundreds of samples (Stepanov et al., 2019).

193 ethnic groups were self-reported in Russia, according to the 2010 census (Russian Census., 2022). The population of Russia’s European part is genetically diverse (Balanovsky et al., 2008; Khrunin et al., 2013; Kushniarevich et al., 2015; Triska et al., 2017). Moreover, self-identified Russians have different ancestry. Specifically, Russians from the north-western part of the country are more closely related to the Finnish population than those from the south-western part of Russia, according to principal component analysis (PCA) (Kushniarevich et al., 2015).

Currently, the largest meta-analysis of height and BMI associated variants for PRS calculation was conducted based on data of 700,000 individuals of joint European and United Kingdom ancestry (Wood et al., 2014; Locke et al., 2015; Yengo et al., 2018). PRS calculated based on European ethnic group cannot be unambiguously applied to another. For example, PRS calculated using the United Kingdom’s height biobank are hardly compatible with the Iberian populations in Spain (Duncan et al., 2019). In this study we utilized complete genomes of 8,664 healthy Russian citizens sequenced by “Evogen”, to evaluate whether the PRS calculated using other European ethnic groups applies to the people of distinct Russian populations.

## 2 Materials and methods

### 2.1 Cohort description

The current study used a collection of a total of 11,753 whole genome sequencing (WGS) samples, sequenced in Russia between September 2019 and 28 July 2022.48.3% of the participants were men and 51.74% were women. The average age was 40.4 ± 19.9 years (men −40.1 ± 19.9 years, women −40.6 ± 19.6 years). Peripheral venous blood samples were collected in EDTA tubes (transported under temperature control). All patients provided informed consent for whole blood sampling for research purposes. The study was approved by the local ethical committee of the Endocrinology Research Center and was performed in accordance with the approved guidelines and the Declaration of Helsinki.

### 2.2 Library preparation

DNA extraction was performed by spin column using the Qiagen QIAamp DNA Blood Kit (Cat. No. 51106) from whole blood according to the manufacturer’s protocol. DNA amount was measured fluorometrically with Qubit4 (Thermo Fisher Scientific)/Denovix (DeNovix Inc.). For the subsequent library preparation only genomic DNA of high quality (OD260/OD280 = 1.8–2.0, OD260/OD230 > 2.0) was used. Library preparation was performed with a PCR-free enzyme fragmentation protocol (MGIEasy FS PCR-Free DNA Library Prep Set, Cat. No. 1000013455) using 800–1,200 ng gDNA. The distribution of insert size was 400–600 bp. WGS library preparation was performed both manually and automatically.

### 2.3 Sequencing

Whole genome sequencing was performed using DNBSEQ-G400 (MGI Tech Co., Ltd.) with FCL PE150 (cat. no. 1000012555), FCL PE200 (cat. no. 1000013858), and DNBSEQ-T7, according to the manufacturer’s protocol.

### 2.4 Data processing

Raw fastq files were processed with MegaBOLT (MGI) for quality control, mapping (hg37) and variant calling. Subsequently, individual vcfs were merged *via* bcftools and further processed with PLINK, hail, PRSice-2 and custom R scripts.

Briefly, for analysis of ethnicity, PLINK files were downloaded from external studies (Fedorova et al., 2013; Kushniarevich et al., 2013; Yunusbayev et al., 2015; Triska et al., 2017; Tambets et al., 2018) and merged into single plink dataset. Shared SNPs were extracted from the merged external dataset and used for subsetting our cohorts with subsequent generation of plink files. Multiallelic and non-genotyped sites were excluded from the analysis. Due to the different array platforms used in these studies and the different genotyping efficiencies, only 36,709 SNP sites were successfully genotyped in all individuals. Principal component analysis (PCA) was carried out by importing plink files to hail matrix and applying default hail *hwe_normalized_pca*. Individual PCA impact was estimated by plotting eigenvalues (Supplementary Figure S1A) and the first ten PCAs were used for selected for further usage (available as Supplementary Table S1). Samples were clusterized based on Euclidean distance between first 10 PCAs using hierarchical clusterisation implemented in R function hclust. Optimal number of clusters for further analysis was selected manually based on evaluation of clusterization accuracy by adjusted mutual information (AMI) which is applicable for evaluation of unbalanced clusterization (Romano et al., 2016) (Supplementary Figure S1B). Ethnic information from external datasets was used as a ground truth labels.

We performed quality control of the target dataset prior to PRS calculations. Firstly, we selected samples with age between 20 and 60 years. Secondly, we calculated the F statistic of heterozygosity rates using PLINK software and removed samples with more than 3 standard deviation (SD) units from the mean. To avoid gender mislabeling and poor quality samples we filtered out females with obtained F statistic for X chromosome homozygosity estimate > 0.2 and males with < 0.8. Then the relatedness of the samples was calculated according to standard PLINK procedure. Samples with relatedness > 0.125 were omitted from the dataset. SNP used for PRS calculation were taken from the corresponding summary statistics. The SNPs with minor allele frequency less than 0.01 and *p*-value of Hardy-Weinberg Equilibrium Fisher’s exact test less than 10^−6^ were used for the further analysis. A total of 8,589 samples passed the filtration and were used for PRS tests.

For PRSs calculation PRSice-2 (Choi and O’Reilly, 2019) was used and the stringent COJO set of summary statistic published by Yengo et al. (2018) as a reference input. Target SNPs were extracted from our cohort (Supplementary File S1) and converted into plink format. Height and BMI were scaled using zscore [phenotype—mean (phenotype)]/sd (phenotype) for each cluster individually and separately for men and women thus eliminating sex specific bias in the phenotypes. Age, sex and the first 10 PCAs were used as a covariate in the model. PRSice was run on the resulting plink files with the following parameters: -stat BETA--beta--binary-target F--target selected_snp_set--bp POS--A1 Tested_Allele--A2 Other_Allele--thread max--cov all. cov--ultra--chr CHR--snp SNP--keep-ambig--no-clump--seed 1215374327. Statistical difference between clusters stratified by PRS was accessed by one way ANOVA. Confidence interval for R2 was calculated by bootstrapping in R boot. ci (type = “bca”) (Davison and Hinkley, 1997; Canty and Ripley, 2022). Quantile plots were generated by PRSice-2.

## 3 Results

### 3.1 The cohort used in the study was of a good quality and suitable for population analysis

**FIGURE 1:**
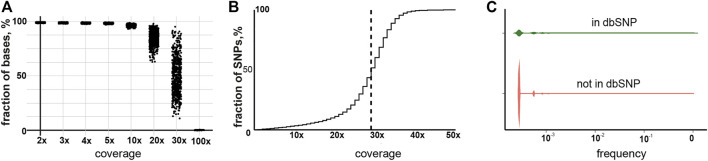
Characterization of the sequencing data and called SNPs. **(A)** Cumulative coverage for samples in the current study. **(B)** Cumulative coverage for called SNPs. The dotted line indicates median coverage. **(C)** Frequency of previously reported and non-reported SNPs.

For the study, we used the large WGS data collection assembled by “Evogen” during screening of the Russian population for carriers of rare genetic diseases. A total of 11,753 samples were taken into initial analysis. In the first step, the overall quality of the WGS data was estimated. Mean sequence depth is one of the most important parameters for robust SNP identification. On average, 90% of the bases were covered with more than 10 reads, which is a sufficient depth for calling germline variations in WGS (Ajay et al., 2011; Kishikawa et al., 2019) (Figure 1A). Indeed, 96.6% of all detected SNPs were covered by more than 10 reads (Figure 1B). We also estimated the amount of SNPs in our dataset, which was already included in the dbSNP database. As expected, the majority of the called SNPs, 64%, were already identified previously and the observed frequencies of the newly described variants in our cohort were significantly lower than ones already reported (Figure 1C). We also have checked the correlation of the observed SNPs frequencies between our cohort and the GnomadV2 database. The Pearson correlation was 0.996 compared to the non-Finnish European population, which is the closest to the Russian population. The overall validations carried out indicated good data quality and applicability for further research.

### 3.2 The population analysis of the cohort revealed presence of the ethnic minorities

The cohort collected across Russia includes ethnically diverse people, and their stratification is crucial for properly assessing the accuracy and predictive power of the PRS. Analyzed cohort did not have meta-information on ethnicity, so we evaluated their population structure using publicly available data for different European and Russian subpopulations (Fedorova et al., 2013; Kushniarevich et al., 2013; Yunusbayev et al., 2015; Triska et al., 2017; Tambets et al., 2018). A principal component analysis (PCA) of the genotype of people from the cohort in combination with several published and publicly available datasets with assigned ethnic information is shown in Supplementary Figure S2. The raw data used to create Supplementary Figure S2 can be found in Supplementary Table S1. The results of the assignment of genotypes from the sample to ethnic groups are shown in Supplementary Table S2. Clusterization was based on the Euclidean distance in the space of the first 10 PCs. Number of clusters selected manually based on AMI Supplementary Figure S1. As was to be expected, the majority of individuals in the collected cohort were in close proximity to previously published populations from central Russia (CR) and neighboring Baltic and Slavic countries. Nevertheless, our randomly sampled cohort included people from many different Russian populations, such as Tatars, Bashkirs, Buryats and others, which underlines the overall ethnic complexity.

### 3.3 The predictive power of PRS for BMI and height is similar for the populations studied

**FIGURE 2:**
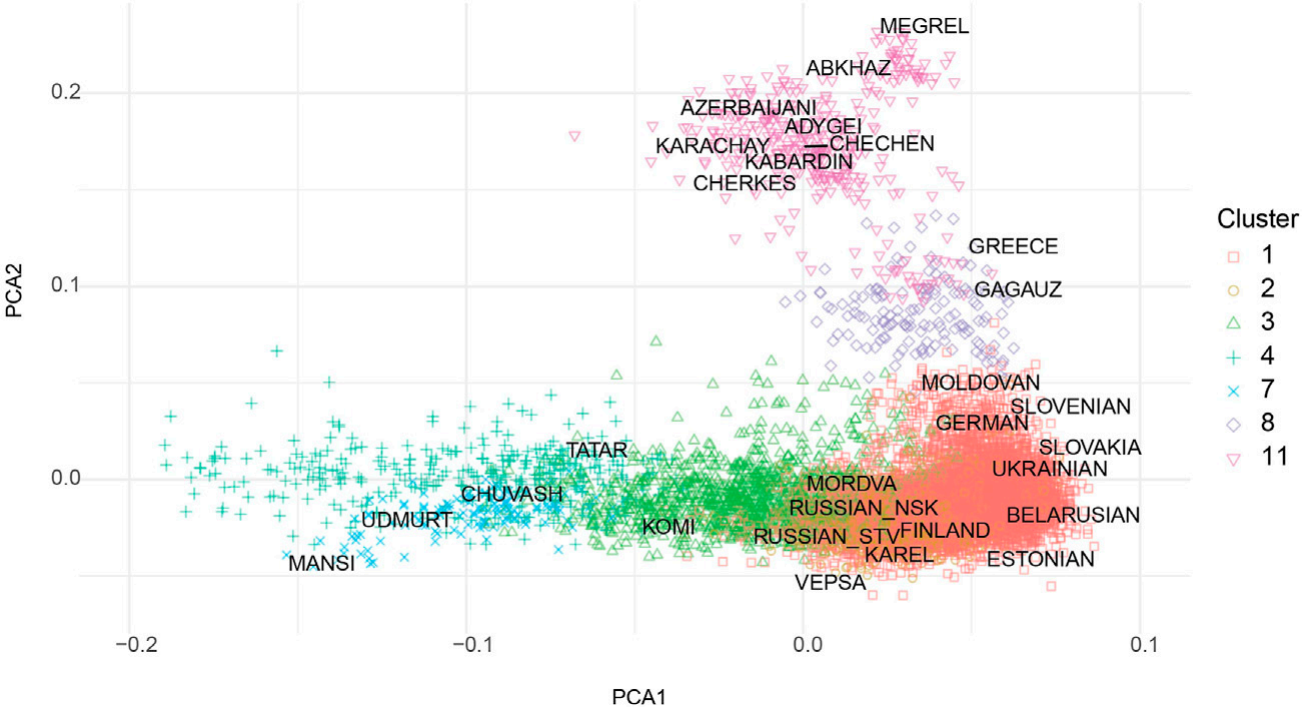
Visualization of first two PCA components obtained from analysis of 39 thousands of shared SNPs for our cohort, selected for PRS calculation (N1 = 5,858, N2 = 992, N3 = 865, N4 = 260, N 7 = 99, N8 = 100, N11 = 203).

**FIGURE 3:**
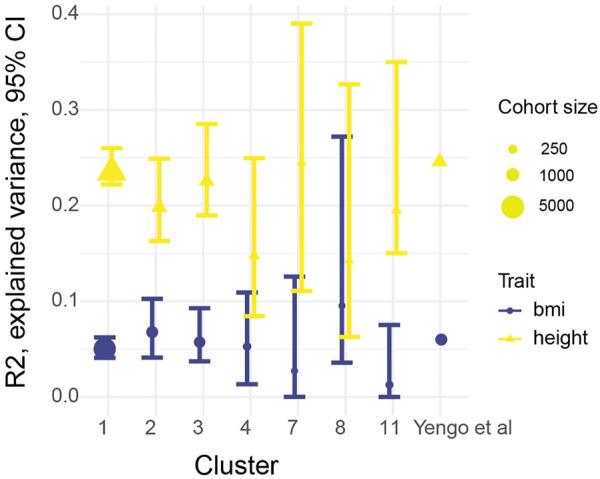
Explained variance (*R* ^2^) produced by PRScise, and 95% bootstrap CI. Dot size represent number of individuals in the our clusters, Yengo et al. (2018) cohort contains 700 thousands individuals.

According to Choi et al. (Choi and O’Reilly, 2019) at least 100 individuals are required for PRS evaluation, therefore clusters with less number individuals were excluded from the further analysis. Resulting cohort presented on Figure 2. Height and BMI distribution for selected clusters presented on Supplementary Figure S3. Summary statistics from the largest, to our knowledge, meta-analysis of variants associated with height and BMI (Yengo et al., 2018) was used to calculate PRS. The most stringent sets of SNPs (3,263 for height and 939 for BMI) reported by the authors were used for the analysis. These SNPs were assumed to be independent from each other based on conditional and joint association analysis (Yang et al., 2011) with a *p*-value cutoff of 10^−8^. The corresponding individual level genetic data file available as Supplementary File S1 and the corresponding phenotypic description can be found in Supplementary Table S2. At the level of SNP frequencies we observed small but significant differences between clusters compared to non-Finish European frequencies from gnomad v2 (Supplementary Figure S4). The PRS for each individual in our cohorts was calculated using PRSice-2 (Choi and O’Reilly, 2019), with an age, sex and the first 10 PCAs being used as a covariate in the model. PRS stratification is shown on Supplementary Figures S5A, B. We found no statistically significant differences between zscores in different PRS strata, by one-way ANOVA. Quantile plots for height and bmi are presented on Supplementary Figures S5C, D. The resulting R2 for height and BMI for each cluster with 95% confidence intervals (CI) is shown on Figure 3; Supplementary Table S2. It should be noted that the PRS calculated on the basis of the European population was applicable to all seven tested clusters of ethnic groups in Russia.

## 4 Discussion

In this work, we tested the relevance of PRS, which was developed based on the European population, on three genetically distinct populations from Russia. It is widely recognized that European ancestry is overrepresented in the majority of publicly available databases (Duncan et al., 2019). At least some Russian populations are closely related to Europeans, which should allow cross application of GWAS summary statistics. Nevertheless, such cross application have not been demonstrated systematically. Recently, Kolosov et al. (2022) have shown that PRS is consistent between northwestern Russians (*n* = 230) and the British population. This result is in concordance with our findings showing that the estimates from PRS, based on the European data, can be applied to different Russian populations. The northwestern Russians belong to population, denoted in the current paper as CR. It is widely accepted that European ancestry is overrepresented in the majority of publicly available databases (Duncan et al., 2019). However, European ancestry in itself is very heterogeneous. This is evident at many levels, including the comparison of SNP frequencies for the different European subpopulations (Lek et al., 2016). These discrepancies may cause PRS incompatibility, such as between the United Kingdom biobank and other European populations, such as the Spanish Iberians or the Italian Tuscans (Duncan et al., 2019). The cohorts analyzed and presented herein substantially surpass previously published data for the Russian population. To the best of our knowledge, the most representative study of genome-wide variation of the Russian people analyzed 263 genomes from 55 ethnic groups (Zhernakova et al., 2020). These data were collected by the Genome Russia Project (*n* = 60), but also by Pagani et al. (2016) (*n* = 173) and Mallick et al. (2016) (*n* = 31). The current study is based on 8,589 individuals for whom individual level genotypes were made publicly available for a set of 4,319 SNPs associated with the inheritance of height and BMI.

The cohort was divided into ethnically different subgroups. The PCA plot is almost identical to the recently published meta analysis of whole exome sequencing of the Russian population by Barbitoff et al. (2021) (Figure 2). That strongly supports our strategy for stratification of individuals into separate cohorts. Unfortunately, even in reasonably large, randomly selected Russian population majority of samples (approximately 70%) fall into the compact single cluster, which represents western Russia inhabitant leaving minor ethnicities underrepresented in our analyses. Nevertheless we were able to gather seven populations with sufficient number of individuals for the analysis.

To accurately assess the transferability of external summary statistics to the selected populations, a selected set of near-independent SNPs with high genome-wide significance (*p* < 10^-8) which were used by (Yengo et al., 2018). The high level of significance was chosen to prevent *R* ^2^ overestimating. Claimed predictive power of the score was reproduced for both phenotypes with narrow CI intervals for the first 3 clusters (Figure 3), which is explained by the large cohort sizes and overall higher resemblance to the European population, judging by overall clusterization (Supplementary Figure S2). For other four populations definitive conclusion could not be drawn from presented analysis, due to apparently insufficient cohort sizes and, therefore large uncertainty in *R* ^2^ estimation. Many things could affect the cross application of summary statistics (Cai et al., 2021) such as admixture in the cohorts, differences in linkage disequilibrium between populations and differences in epistasis. Nevertheless, given that *R* ^2^ for height was comparable between all three cohorts and one previously published, we might speculate that the observed decline in *R* ^2^ for BMI is not related to differences in genetics but rather to differences in environmental factors such as different cultural background and diet.

It is worth noting that recapitulation of predictive results does not lead to recapitulation of the PRS distribution across the whole population (Duncan et al., 2019). Therefore, the PRS distribution for individual strata for risk management must still be assessed separately (Qassim et al., 2021).

The driving force behind the cross application of PRSs between populations comes from the successes in predicting the risk of diseases with strong genetic components. Genome-wide association studies (GWASes) have revealed the complex nature of common disease pathogenesis (Burton et al., 2007). The association between human genome variants and disorders has been demonstrated for bipolar disorder (Stahl et al., 2019), rheumatoid arthritis (Smolen et al., 2018), type 1 (Bonifacio et al., 2018) and type 2 (Montesanto et al., 2018) diabetes mellitus, coronary artery disease (Erdmann et al., 2018) and other pathologies. We consider this work as a proof of concept for the cross application of PRS developed based on European cohorts to Russian populations described here.

## Data Availability

The data presented in this study is available in the Supplementary Material.

## Supplementary material

Supplementary Figure S1 Clusterization characteristics. Elbow plot **(A)**, adjusted mutual information **(B)**, vertical line represents manually selected number of clusters.

Supplementary Figure S2 Visualization of first two PCA components obtained from analysis of 39 thousands of shared SNPs for our population (*N* = 11753) and external studies (*N* = 1,353). Colors represent clusterization into a predefined number of clusters (28) in the space of first 10 PCAs based on Euclidean distance.

Supplementary Figure S3 Height **(A)** and BMI **(B)** distribution for selected cohorts.

Supplementary Figure S4 Comparison of frequencies of SNPs from summary statistics published by Yengo et.al. (2018) in our cohorts and Non-Finish Europeans.

Supplementary Figure S5 Correlation between a trait (z-scored) and PRS, splitted into deciles for height **(A)** and BMI **(B)**.

Supplementary Table S1 First 10 PCAs used for clusterization provided on Supplementary Figure S1.

Supplementary Table S2 Phenotypic description of the cohort used in the study.

Supplementary File S1 Multisample vcf file with SNPs used for PRS calculation.
