# Supplementary figures and images for: Transferability of the PRS estimates for height and BMI obtained from the European ethnic groups to the Western Russian populations
Source: Front Genet. 2023 Jan 16;14:1086709. doi: 10.3389/fgene.2023.1086709 (PMC9885218; doi:10.3389/fgene.2023.1086709)

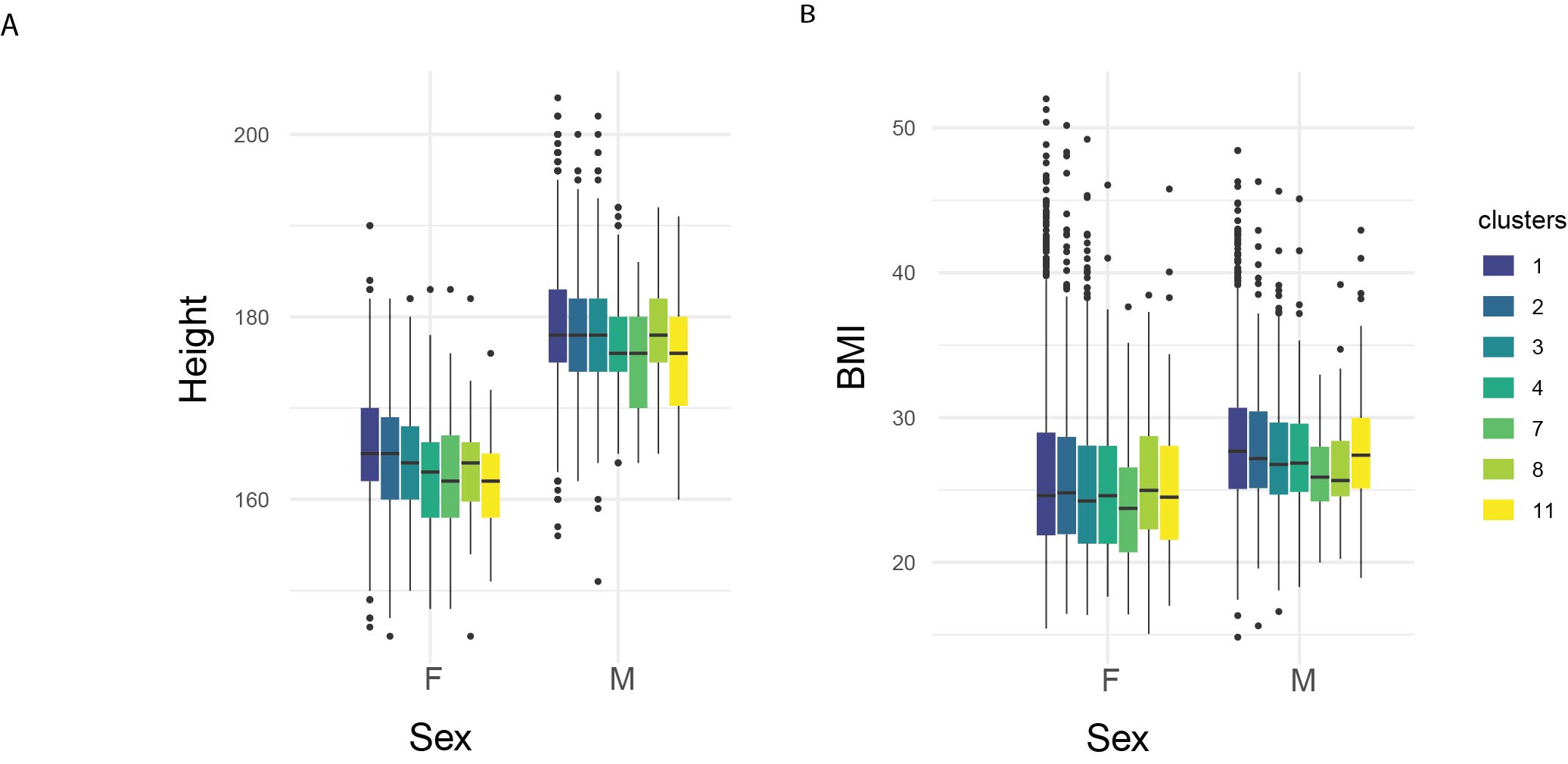

Supplement: Supplementary file 2 [file Image3.JPEG]

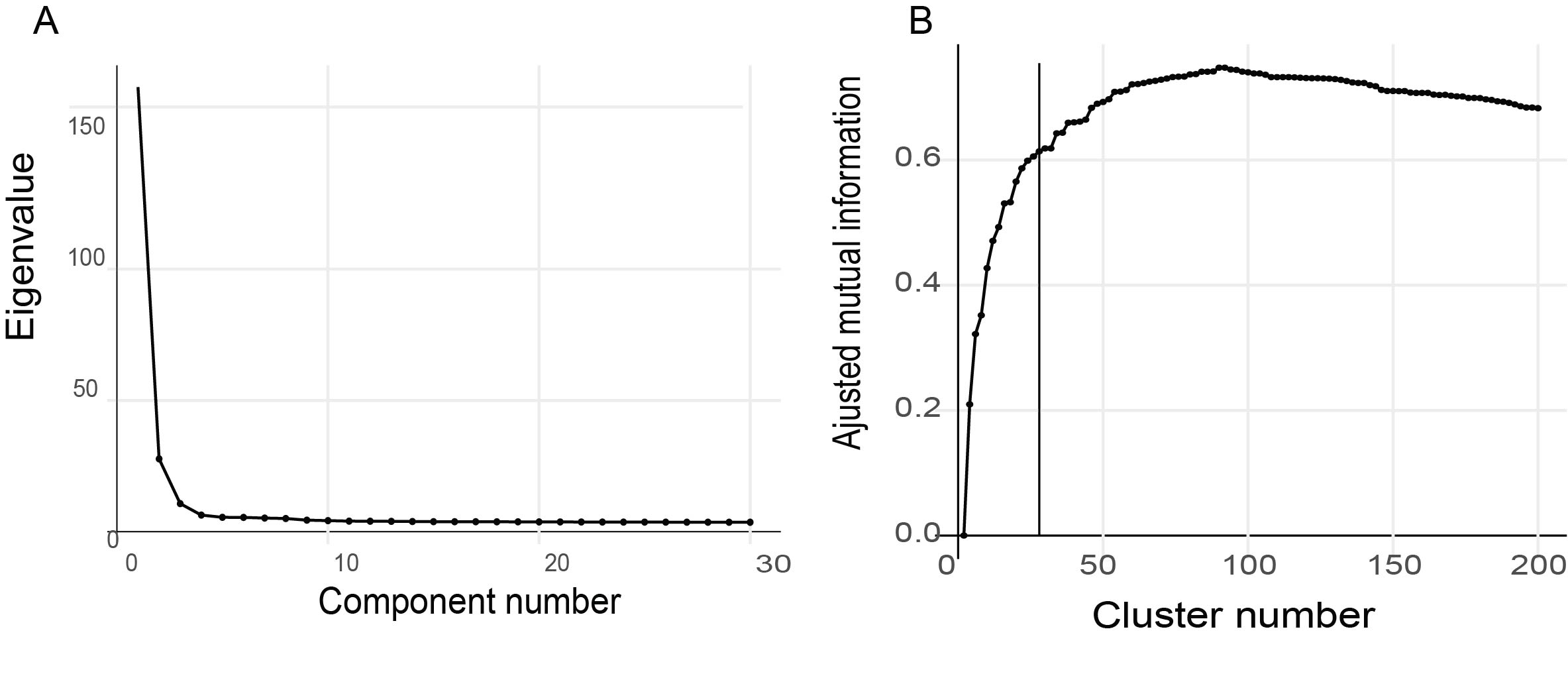

Supplement: Supplementary file 3 [file Image1.JPEG]

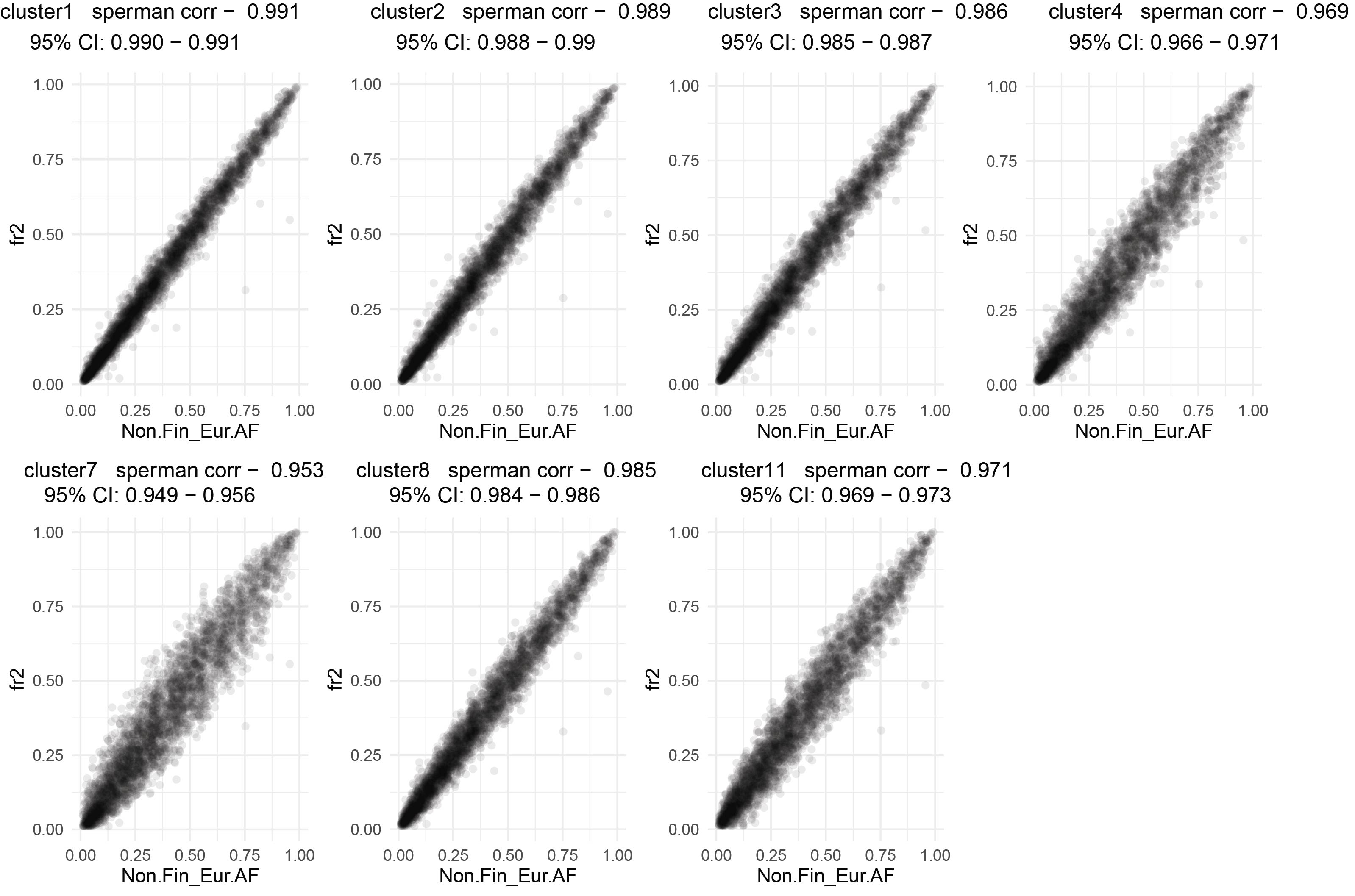

Supplement: Supplementary file 4 [file Image4.JPEG]

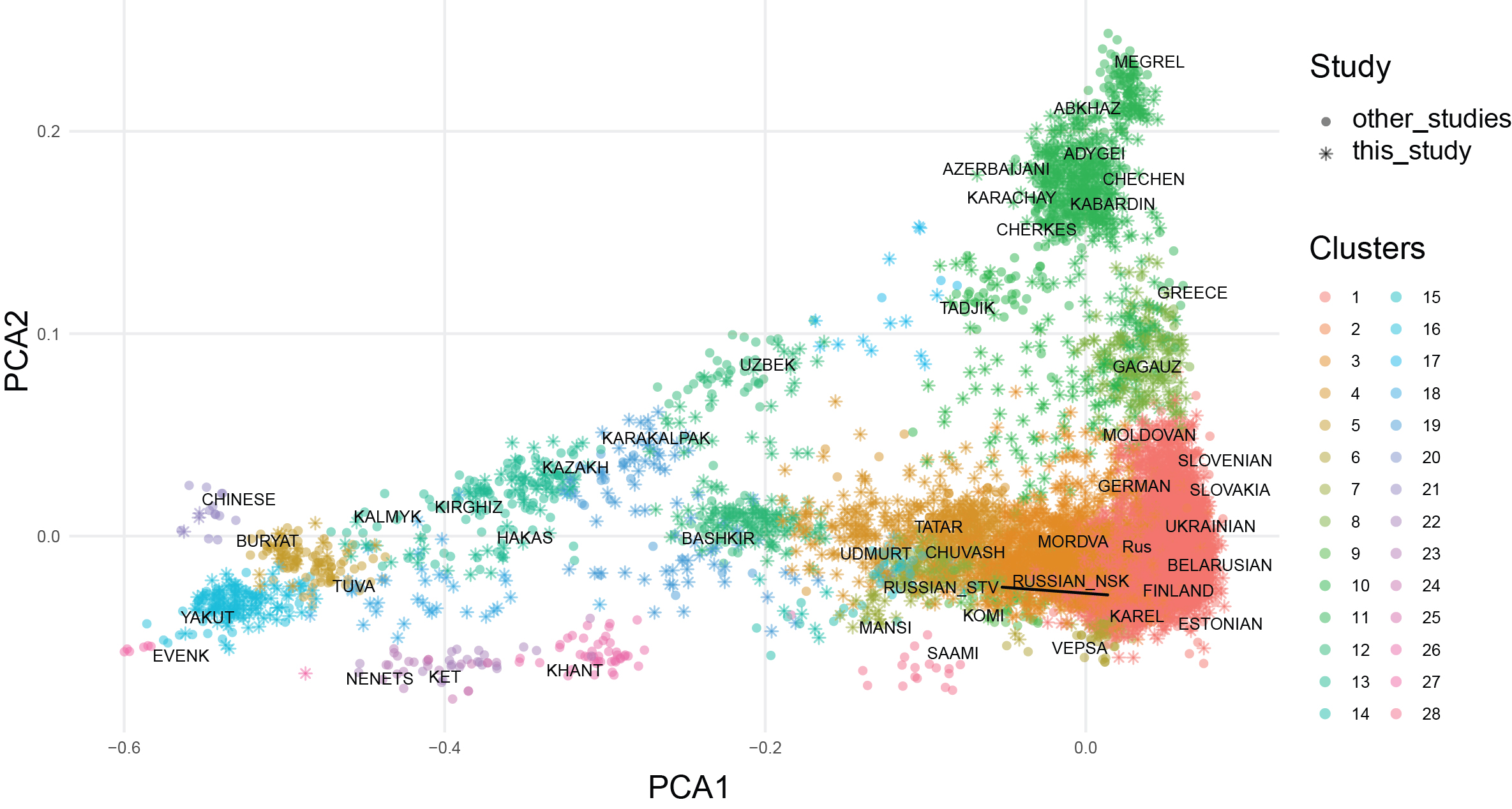

Supplement: Supplementary file 6 [file Image2.JPEG]

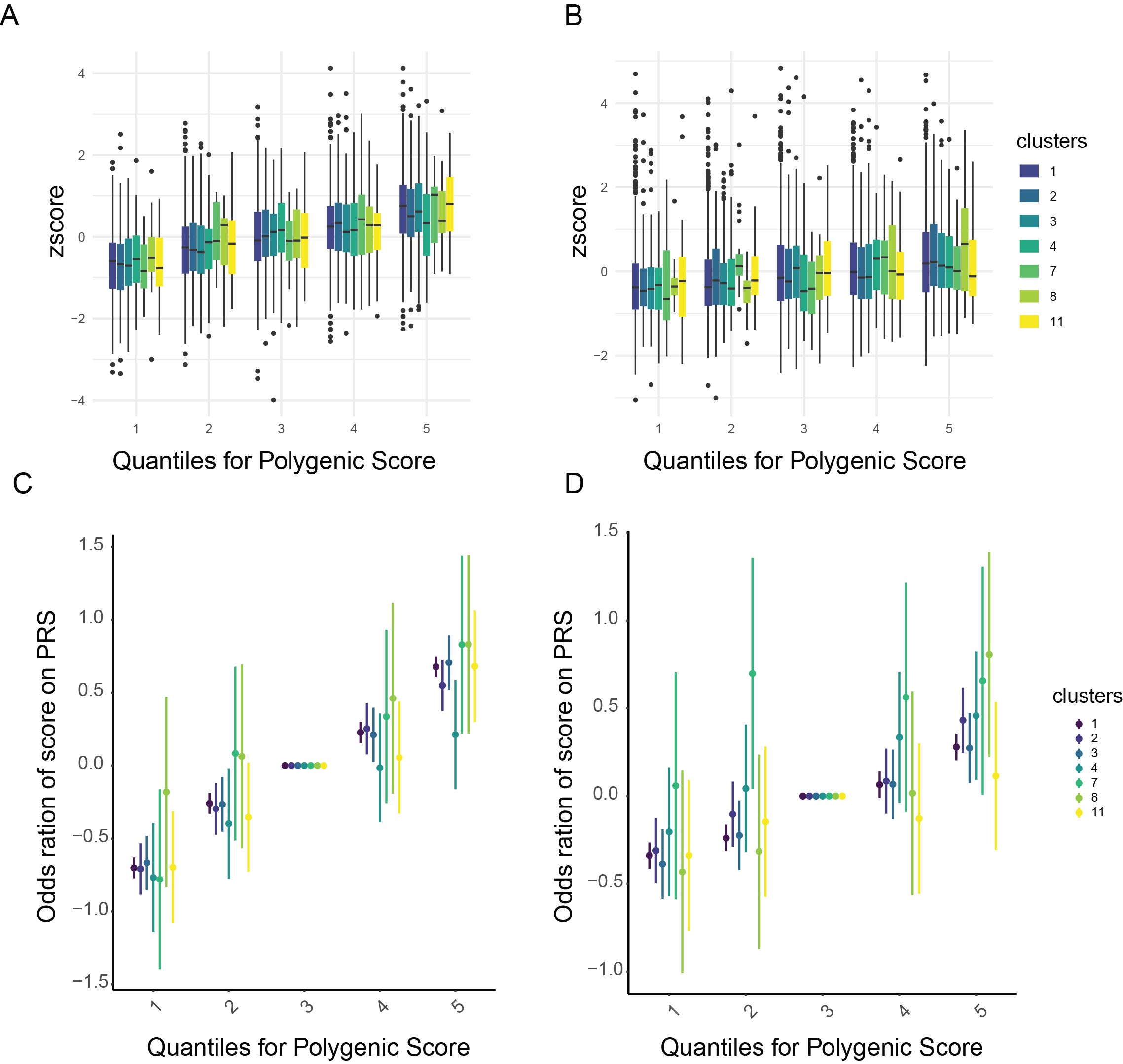

Supplement: Supplementary file 7 [file Image5.JPEG]
